# Internally coupled ears in living mammals

**DOI:** 10.1007/s00422-015-0675-1

**Published:** 2016-01-22

**Authors:** Matthew J. Mason

**Affiliations:** Department of Physiology, Development and Neuroscience, University of Cambridge, Downing Street, Cambridge, CB2 3EG UK

**Keywords:** Middle ear, Mole, Golden mole, Platypus, Hearing, Pressure-difference receiver

## Abstract

It is generally held that the right and left middle ears of mammals are acoustically isolated from each other, such that mammals must rely on neural computation to derive sound localisation cues. There are, however, some unusual species in which the middle ear cavities intercommunicate, in which case each ear might be able to act as a pressure-difference receiver. This could improve sound localisation at lower frequencies. The platypus *Ornithorhynchus* is apparently unique among mammals in that its tympanic cavities are widely open to the pharynx, a morphology resembling that of some non-mammalian tetrapods. The right and left middle ear cavities of certain talpid and golden moles are connected through air passages within the basicranium; one experimental study on *Talpa* has shown that the middle ears are indeed acoustically coupled by these means. Having a basisphenoid component to the middle ear cavity walls could be an important prerequisite for the development of this form of interaural communication. Little is known about the hearing abilities of platypus, talpid and golden moles, but their audition may well be limited to relatively low frequencies. If so, these mammals could, in principle, benefit from the sound localisation cues available to them through internally coupled ears. Whether or not they actually do remains to be established experimentally.

## Introduction

If sound can reach a tympanic membrane both directly from the external environment and also from the contralateral ear, via an internal acoustic pathway, the membrane will respond to the instantaneous difference in pressures applied to its external and internal surfaces. This results in a directional tympanic membrane response which can increase both interaural time and amplitude differences over a certain frequency band, improving the sound localisation abilities of the organism in question (Christensen-Dalsgaard 2011; Köppl 2009). The terminology used to refer to these phenomena can vary: in this paper, the following definitions are used. If sound transmitted into one middle ear can somehow pass through the head and reach the inside of the contralateral tympanic membrane, the animal is said to have *internally coupled ears*. A tympanic membrane thus exposed to acoustic pressures on both sides acts as a *pressure-difference receiver*. Sound localisation which makes use of pressure-difference receivers is referred to as *pressure-difference sound localisation*.

Non-mammalian tetrapods with tympanic ears often have relatively wide air channels connecting left and right middle ear cavities. Such connections are most obvious in frogs and many lizards, in which middle ear cavities communicate widely with the mouth, while in birds and crocodilians the middle ear cavities intercommunicate by means of air pathways extending through the bones of the skull (Baird 1970; Henson 1974; Saunders et al. 2000; Wever 1985 represent good anatomical reviews). Given these anatomical connections, pressure-difference sound localisation is likely to be widespread among non-mammalian vertebrates, a conclusion supported by experimental studies of amphibians, birds and lizards [reviewed by Christensen-Dalsgaard (2011), Christensen-Dalsgaard and Carr (2008) and Manley (2010)]. Alligators have recently been added to the list of animals believed to localise sound in this way (Bierman et al. 2014). Extant turtles are exceptional in having enclosed middle ears with narrow Eustachian tubes, which may be related to their mechanism for sound reception underwater (Christensen-Dalsgaard 2011; Willis et al. 2013).

Like turtles, mammals typically have narrow Eustachian tubes. Mammalian middle ear cavities are, in many species, enclosed within separate bony swellings on the ventral basicranium known as the auditory bullae. There are no air pathways linking the two middle ears, except when the Eustachian tubes are open. The acoustic independence of the middle ears in mammals brings several benefits, notably protection, given that they are not in wide communication with the pharynx, and the fact that isolated ears working as simple pressure receivers tend to have better low frequency responses than pressure-difference receivers (Christensen-Dalsgaard 2011; Christensen-Dalsgaard and Carr 2008). However, without pressure-difference sound reception, mammals must rely on neural computation to extract localisation cues from sound received separately on the right and left sides of the head (Manley 2010). Pinnae can facilitate sound localisation by increasing interaural time and level differences (ITD, ILD), and by introducing monaural spectral cues (Koka et al. 2011, 2008). Even so, in order to localise sound effectively, mammals require higher-frequency hearing than species with internally coupled ears. The problem is particularly acute in small mammals because of small time-of-arrival differences between the closely set ears, and reduced sound shadowing by the head (Heffner and Heffner 1992a; Köppl 2009). Köppl (2009) gives the rule-of-thumb that usable ILDs would only be available to a mouse-sized mammal at frequencies over 10 kHz, which is, coincidentally, more-or-less the upper limit of hearing for non-mammalian vertebrates. Hearing ranges in mammals tend to extend to much higher frequencies: the hearing of the house mouse *Mus*, for example, extends to around 90 kHz at 60 dB SPL (Heffner and Masterton 1980). The smaller the interaural distance, the higher the frequencies necessary for sound localisation, which is presumably why the upper limit of hearing in mammals correlates inversely with interaural distance (Heffner and Heffner 1992a).

To summarise, mammals are generally regarded as having acoustically isolated middle ears, which means that in order to localise sound effectively they require, and have, excellent high-frequency hearing compared with non-mammals of similar body size. Is this true of *all* mammals though? The present paper considers the question of whether pressure-difference sound localisation could be possible in any mammalian species. Experimental evidence addressing this issue remains very limited, so the approach taken here is (1) to consider anatomical pathways between the right and left middle ears of mammals which might result in significant acoustic coupling, and (2) to consider whether mammals possessing such pathways would require directionally sensitive tympanic membranes in order to localise sound sources. From the arguments presented above, pressure-difference sound localisation is most likely to benefit a small mammal with hearing restricted to low frequencies, particularly if it lacks pinnae. I shall argue that, based on the evidence available, the mammalian species most likely to use a form of pressure-difference sound localisation are talpid moles, golden moles and the platypus.

## Material and methods

Micro-computed tomography (micro-CT) scans were made of the preserved heads or prepared skulls of several species of small mammals. One specimen of the talpid mole *Talpa europaea* was obtained as a frozen corpse: it had originally been trapped as a pest in Essex, UK. One specimen of the shrew *Sorex araneus* was found dead in Cambridgeshire, UK: its head was preserved in 75 % ethanol. The skinned heads of both of these specimens were wrapped in cellophane prior to scanning. An ethanol-preserved head of the sengi *Macroscelides flavicaudatus* (CAS MAM 30152) had been obtained on loan from the collection of the Department of Ornithology & Mammalogy, California Academy of Sciences, San Francisco, and had been scanned as part of another study (Mason 2016a). Prepared museum skulls of the golden moles *Amblysomus hottentotus* and *Chrysochloris asiatica* had been obtained from the Transvaal Museum, Pretoria, for use in earlier studies (Mason 1999, 2003). CT scans of all these specimens were made at the Cambridge Biotomography Centre using a Nikon XT H 225 scanner; the settings were 120–130 kV and 110–120 $\upmu$A. Tomograms were constructed from 1080 projections, each with 1000 ms exposure and two frames averaged per projection. The software used in the processing of the scan data included CT Agent XT 3.1.9 and CT Pro 3D XT 3.1.9 (Nikon Metrology, 2004–2013). Cubic voxel side lengths were 11–20 $\upmu$m.

Further new data were obtained by reprocessing and reanalyzing a previously unpublished CT scan of a prepared skull of the talpid mole *Scaptochirus moschatus* (spec. no. 1928.1.6.2), obtained on loan from The Natural History Museum, London. The scan had been made in 2005 in the Department of Engineering, University of Cambridge, using a Metris X-Tek HMX 160 micro-CT scanner, with settings of 65 kV and 75 $\upmu$A. Tomograms were constructed from 360 projections, with 32 frames averaged per projection. The software used in the reprocessing of the data included iXS Integrated X-ray System Control version 4.1.29 (X-Tek Systems Ltd., 2002), NGI CT Control version 1.5.4 (X-Tek Systems Ltd., 2005) and CT-Pro 2.0 (Metris, 2008). Cubic voxel side lengths were 45 $\upmu$m.

Tomograms were converted to jpeg format using Adobe Photoshop CS 8.0 (Adobe Systems Inc., 2003). 3D reconstructions of skulls and reorientations of section planes were made with Microview 2.1.2 (GE Healthcare, 2000-6). WinSurf 4.0 (Moody and Lozanoff 1998) was used to construct three-dimensional images of ear structures, following manual tracing of their borders, and cavity volumes were established from these using the same software.

## Potential routes for internal coupling of mammalian ears

**Fig. 1:**
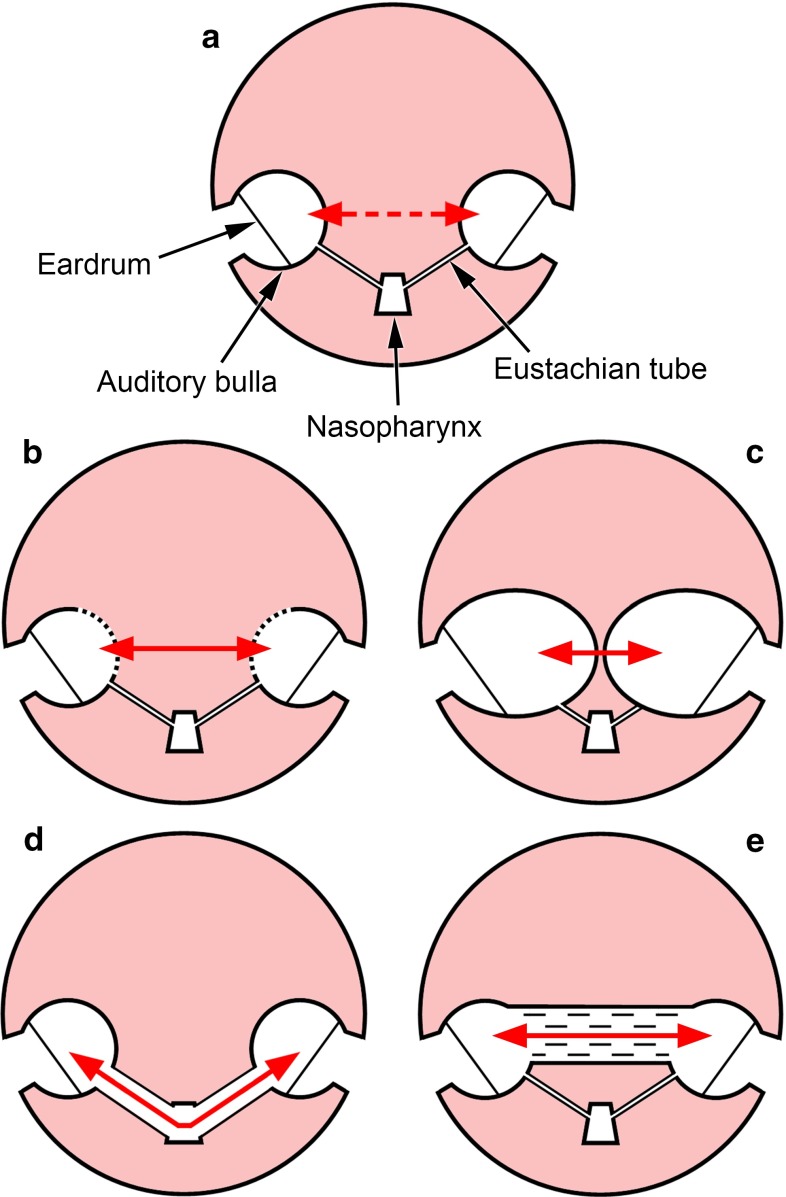
Diagrammatic transverse sections of the heads of mammals, illustrating some of the pathways by which the left and right middle ear air cavities might potentially be acoustically coupled (*red arrows*). The middle ear cavities are contained within the bony auditory bullae. **a** In a typical mammal, the two auditory bullae are well separated, each being connected to the nasopharynx through a narrow Eustachian tube which is collapsed most of the time. Bone conduction through the skull may occur in “cross-hearing” experiments, but the bullae are normally considered to be acoustically isolated with respect to airborne sound stimulation. **b** In species with incomplete bullae such as shrews, sound might enter or leave each middle ear cavity through non-ossified regions of the walls, passing through the intervening soft tissues of the head. **c** In species with very large bullae such as *Macroscelides*, sound transmission between the ears may be possible where the bullae converge at the midline. **d** Air pathway created by wide, patent pharyngo-tympanic connections, as in *Ornithorhynchus*. **e** The middle ear cavities in some talpid and golden moles are coupled via air pathways passing through the basicranial bones of the skull

The most obvious ways in which right and left middle ears could in principle be acoustically coupled in mammals include (1) bone conduction through the skull, (2) soft tissue conduction via incomplete middle ear cavity walls, (3) the juxtaposition of bullae in the midline, (4) wide, patent Eustachian tubes connecting each middle ear to the pharynx, or (5) air passages within the basicranial bones of the skull. These possible pathways are illustrated diagrammatically in Fig. 1 and will be considered in turn.

### “Crossover stimulation” by bone conduction

Although the right and left ears of most mammalian species are well separated, interaural sound transmission is still possible (Fig. 1a). For example, if loud sound is introduced into a damaged ear through an earphone, the contralateral (undamaged) ear receives some sound input too. Its response to this, referred to as “crossover stimulation” or “cross-hearing”, is clinically significant because it may affect the results of a test designed to assess the hearing loss in the damaged ear (Brännström and Lantz 2010). The reduction in sound level between the ipsilateral ear, to which the sound is applied, and the contralateral ear is referred to as the interaural attenuation.

Interaural attenuation in mammals has been measured in several different ways. Teas and Nielsen (1975) presented sound to one ear of a chinchilla at frequencies from 0.3 to 14 kHz, and compared cochlear microphonic responses from the ipsi- and contralateral ears. With the bullae sealed, they found an attenuation of 40–72 dB across the head. Similar results had previously been obtained by Mast (1970). More recently, Arnold and Burkard (2000) demonstrated interaural attenuation of 40–85 dB in chinchillas by comparing evoked potential thresholds from the inferior colliculus when sound (0.5–8 kHz) was applied either to a normal ear or to the contralateral ear, within which the cochlea had been destroyed. The amount of interaural attenuation observed in mammals depends on the species considered, the sound frequencies used and, crucially, the mechanism of sound delivery (Arnold and Burkard 2000; Megerian et al. 1996). It is typically large, however, a minimum attenuation of around 40 dB having been measured under differing experimental circumstances in guinea pigs (Teas and Nielsen 1975), cats (Gibson 1982) and humans (Brännström and Lantz 2010; Zwislocki 1953), and 30 dB in rats (Megerian et al. 1996), with most specimens showing much greater values at most frequencies.

In these experiments, sound is usually delivered to one ear using a closed system, such as an insert earphone which fits snugly into the external meatus and helps to prevent airborne sound from leaking out. Sound from the earphone will excite the ipsilateral middle ear, resulting in an auditory response in the normal way if that ear is intact, but earphone vibrations will inevitably set the skull into vibration too. Skull vibrations have been shown to pass from one side of the human skull to the other with very little attenuation, especially at low frequencies (see e.g. Stenfelt 2012), and can excite the contralateral cochlea through a variety of pathways collectively referred to as bone conduction (Stenfelt and Goode 2005). Bone conduction is believed to represent a major component of interaural sound transmission in earphone experiments (Megerian et al. 1996; Zwislocki 1953).

We are interested here in the question of whether interaural coupling is possible in mammals to an extent sufficient to drive pressure-difference sound reception. Outside of the laboratory, the sound reaching the ears of most mammals is usually travelling in air. Diffuse-field airborne sound will vibrate the skull directly and can drive bone-conducted hearing, albeit with high thresholds in humans (Reinfeldt et al. 2007). For interaural coupling to exist, the vibrations of one middle ear, in response to airborne sound, would have to induce bone-conducted vibrations of the head of high enough amplitude that the contralateral tympanic membrane would also be excited. No specific experimental data have been found which address this question directly, but it is fair to assume that a middle ear responding to airborne sound will excite the skull much less than a vibrating earphone attached to that ear, in which case the interaural attenuation should exceed the high values reported in the earphone studies discussed above. Furthermore, the amount of bone-conducted sound radiated into the middle ear cavity has been found to be negligible in experiments on cats (Tonndorf et al. 1966a, b) and human temporal bone specimens (Stenfelt et al. 2002). Under such circumstances, pressure-difference sound reception by the contralateral tympanic membrane would be essentially impossible.

The conclusion is that, under normal circumstances, crossover stimulation by bone conduction is negligible, and the two ears are essentially isolated from each other. This is widely (if tacitly) assumed to hold for mammals in general.

### Internal coupling via soft tissue in species with incomplete bullae

It is believed that the common ancestor of living mammals lacked complete auditory bullae, a condition still found in many “primitive” species (Fleischer 1978; Novacek 1977; Rosowski 1992; Zeller 1993). In such cases, the ectotympanic bone supporting the tympanic membrane is not fused with the surrounding bones of the basicranium, but is instead more loosely supported by ligaments and connective tissue. A route for sound energy transfer therefore exists between the two middle ear cavities which involves passage of sound into and out of soft tissue or fluid (Fig. 1b). The attenuation occurring en route, although substantial, might be expected to be less than in the case of an animal possessing middle ear cavities enclosed within complete, bony walls.

Mammals lacking complete bullae include monotremes, some marsupials such as opossums, hedgehogs, shrews, certain talpid moles and tenrecs (Burda 1979; Mason 1999, 2006; McDowell 1958; Novacek 1977; Van der Klaauw 1931; van Kampen 1905; Zaytseva et al. 2015). The ectotympanics of these species are often inclined at near-horizontal angles and are closely apposed to the basicranial bones above them, in some cases overlapping. Because of this, the extent of the non-ossified “windows” in the walls of these middle ear cavities varies considerably between groups. Shrews appear to have the least enclosed cavities.

**Fig. 2:**
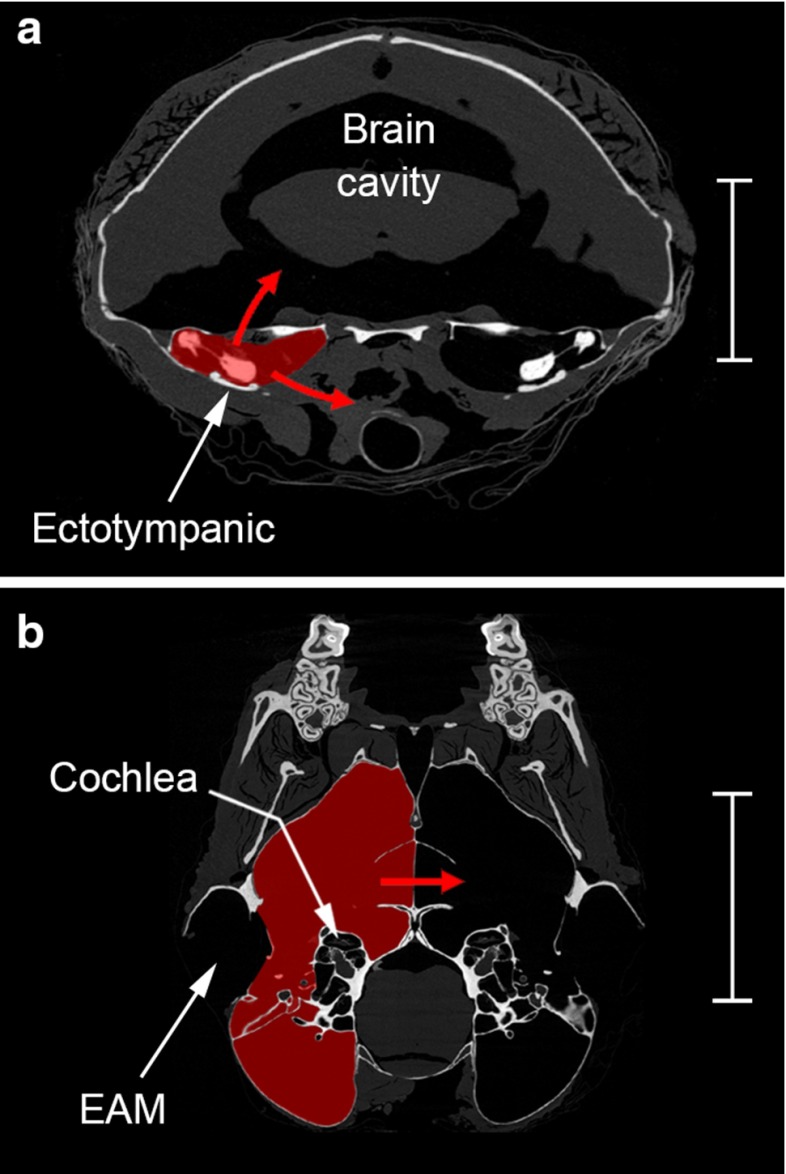
Micro-CT sections through the heads of two small mammals. The middle ear cavities on the *left sides* have been *shaded red* in each case. **a** An approximately transverse plane through the head of the shrew *Sorex araneus*; *scale bar* 3 mm. The ectotympanic in this species is a ring-shaped element which does not form a complete bony bulla; the malleus lies just above this. Potential pathways for sound energy to escape the middle ear cavity through its soft tissue boundaries are indicated as *red arrows*. In shrews, the dorsal roof of the middle ear cavity contains a large, unossified vacuity, so one such route involves the brain cavity. **b** An approximately frontal plane through the posterior part of the head in the sengi *Macroscelides flavicaudatus*; *scale bar* 10 mm. In this animal, the two bullae are so extensive that they share a common wall in the midline: the *red arrow* indicates how sound might in principle reach the contralateral ear through this thin, bony lamina. *EAM* external auditory meatus

The hearing of monotremes may well be restricted to relatively low frequencies for mammals (see Sect. 3.4), while the hedgehog *Erinaceus* was found to respond only to sound within the sonic range (Konstantinov et al. 1987). However, experimental studies of another hedgehog, several shrews, several opossums and a tenrec have revealed that these animals generally have much better high-frequency hearing, extending to 40 kHz or above (Drexl et al. 2003; Frost and Masterton 1994; Konstantinov et al. 1987; Ravizza et al. 1969a, b). Because they are such small animals, the right and left middle ear cavities of shrews are separated by no more than a few millimetres, and the cavities lack bony walls altogether on their medial sides (Fig. 2a). Shrew ears are therefore expected to experience the most significant internal coupling through the soft tissue route, but shrew hearing extends to such high frequencies that there is little reason to suspect that these animals would need to rely on pressure-difference sound localisation.

### Convergence of enlarged bullae at the midline

The bony auditory bullae can be relatively large in some small mammals, to the point where they approach or even touch each other in the midline of the skull (Mason 2016a; Oaks 1967; Van der Klaauw 1931): this raises the possibility of direct acoustic coupling (Fig. 1c). Although Nummela (1995) notes that the middle ear cavities of the chinchilla are “hypertrophied and mutually connected”, no direct communication has been reported in more detailed studies of the chinchilla ear (Argyle and Mason 2008; Browning and Granich 1978; Daniel et al. 1982; Hanamure and Lim 1987; Oaks 1967; Vrettakos et al. 1988). The most extreme case may instead be the sengi *Macroscelides*, in which the two middle ear cavities are separated only by a thin, bony septum in the midline (Mason 2016a; Fig. 2b).

Enlarged bullae in gerbils and chinchillas, which have been well studied, are believed to increase middle ear compliance and thus improve low-frequency sensitivity (Mason 2016b; Ravicz and Rosowski 1997; Rosowski et al. 2006). However, the hearing ranges of these animals still extend to ultrasonic frequencies (Heffner and Heffner 1991; Ryan 1976), and pressure-difference reception has not been implicated in studies of their sound localisation (Heffner and Heffner 1988; Heffner et al. 1994, 1995, 1996; Koka et al. 2011). The hearing of *Macroscelides* has never been directly tested.

### Internal coupling via the pharynx

The embryological connection between middle ear and pharynx is retained in adult mammals as the Eustachian tube. There is always, therefore, a potential air passageway from one middle ear cavity down the Eustachian tube to the nasopharynx, and from there up the contralateral Eustachian tube to the other middle ear cavity. However, unlike the short, wide and patent passageways in frogs and some lizards, the Eustachian tubes of mammals are typically very narrow and their cartilaginous portions remain collapsed for much of the time. Opening is under active, muscular control and occurs in association with actions such as swallowing, periodically equalising static pressures between middle ear cavity and nasopharynx. Bluestone and Doyle (1988) provide a useful introduction.

A collapsed Eustachian tube does not represent a significant conduit for sound but a patent Eustachian tube could, in principle, result in coupling between the ears (Fig. 1d). Patulous Eustachian tube is an uncommon human clinical condition in which the tube remains open continuously: sufferers hear their own breathing, voice and chewing abnormally loudly, and their tympanic membranes may move visibly with ventilation (O’Connor and Shea 1981). Reducing the low-frequency noise generated in this way may have been one driving force behind the narrowing of Eustachian tubes in mammals (Christensen-Dalsgaard 2011; Christensen-Dalsgaard and Manley 2005).

The morphology of the Eustachian tube varies among mammals. Horses, tapirs, hyraxes and certain bats have sac-like diverticula of their Eustachian tubes (Hinchcliffe and Pye 1969; Lechner 1932; Pye and Hinchcliffe 1976; Van der Klaauw 1931), but there has been no suggestion that the ears of these animals are internally coupled. Of more interest to the present discussion, chinchillas have been found to have “semi-patulous” Eustachian tubes, apparently as a normal condition. This was demonstrated in a study of awake experimental animals, in which it proved impossible to maintain middle ear pressure at a different level from atmospheric due to passive leakage through the tube (Doyle 1985). Browning and Granich (1978) observed the tympanic membrane in this species to be very mobile during respiratory ventilation. In contrast, Eames et al. (1975) noted that sedated chinchillas, unlike awake animals, were unable to equalise middle ear pressures. Perhaps muscular tone is needed to keep the tube from collapsing, or perhaps the tube in sedated animals becomes blocked by mucous secretions or, as Eames et al. suggested, tubal oedema. The chinchilla Eustachian tube is around 4.5 mm long, with a narrow luminal width (Hanamure and Lim 1987), so even if fully open it is expected to confer a substantial acoustic impedance. The high levels of interaural attenuation shown to exist by Arnold and Burkard (2000), discussed in Sect. 3.1, were measured in unanaesthetised animals at least 2 weeks after surgery, in which the tubes should have been functioning normally.

**Fig. 3:**
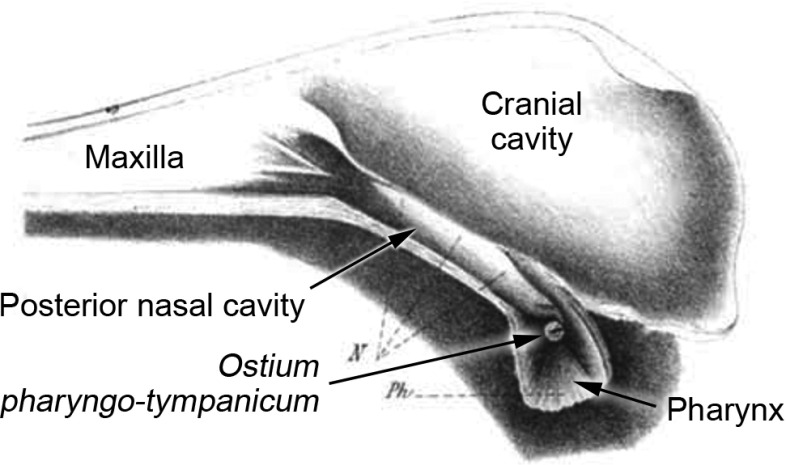
Medial view of the right side of a sagittally sectioned head of the platypus *Ornithorhynchus anatinus*, from Zuckerkandl (1886), relabelled. The *ostium pharyngo-tympanicum* represents the open connection between nasopharynx and tympanic cavity, which in the platypus replaces the narrow Eustachian tube typical of other mammals. Presumably, the *ostium* appears bright due to light from the other side of the head shining through the tympanic membrane. The dark rod within the bright circle is the manubrium of the malleus. Reproduced with kind permission from Springer Science+Business Media: *Archiv für Öhrenheilkunde*, Beiträge zur vergleichenden Anatomie der Ohrtrompete, 23, 1886, 201–213, Zuckerkandl, E., Fig. 1

There is no good evidence, then, that significant internal coupling occurs in any mammal via the narrow Eustachian tubes which are characteristic of this group. There is, however, one mammal which reportedly has a middle ear in wide and open communication with the nasopharynx: the platypus, *Ornithorhynchus anatinus *(Denker 1901; Eschweiler 1899; Zuckerkandl 1886; Fig. 3). Zuckerkandl compared the pharyngo-tympanic connection of the platypus to that of frogs, which are now known to have internally coupled ears (Chung et al. 1981). The very limited information available relating to audition in the platypus suggests that it is rather insensitive and restricted to low frequencies, but testing has not extended above 20 kHz (Gates et al. 1974; Krubitzer 1998). If its hearing is indeed restricted to a low-frequency range, the platypus, which lacks pinnae (Nowak 1999), might well benefit from pressure-difference sound localisation.

Apart from the platypus, the only other living monotremes are the echidnas. Hearing in *Tachyglossus aculeatus* appears similarly restricted to low frequencies (Aitkin and Johnstone 1972; Mills and Shepherd 2001). However, unlike the platypus, *Tachyglossus* has a narrow Eustachian tube resembling that of other mammals (Denker 1901; Eschweiler 1899; Zuckerkandl 1886), and so it is less likely to have internally coupled ears.

### Middle ear coupling via pneumatised basicranial bones

**Fig. 4:**
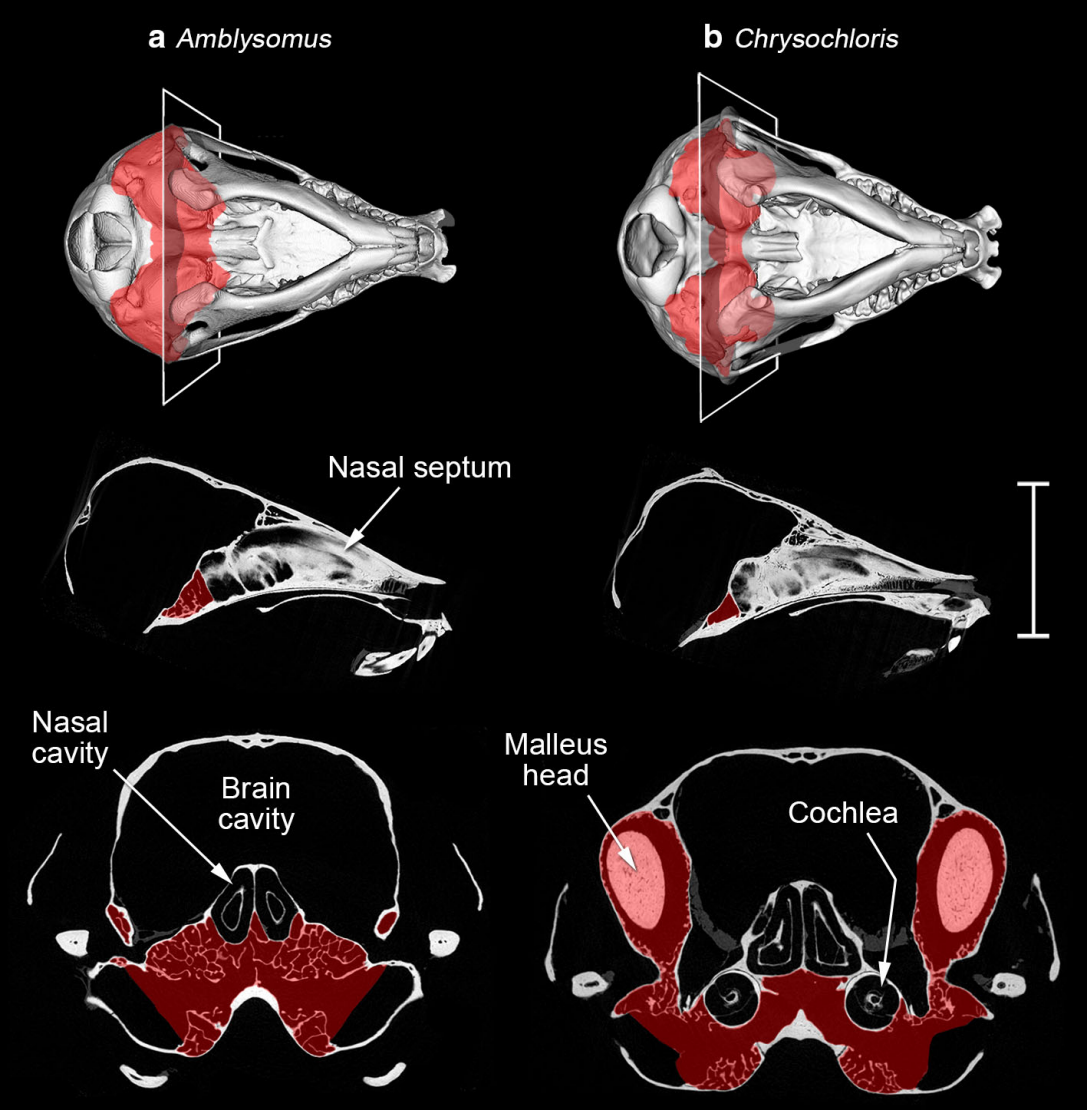
Micro-CT reconstructions of the prepared skulls of two golden moles, **a** *Amblysomus hottentotus*, on the *left*, and **b** *Chrysochloris asiatica*, on the *right*. The *upper row* shows Microview reconstructions of the skulls in ventral view. Damaged areas of the skulls have been replaced with *grey shading*. The planes of the transverse sections shown below are indicated. The *middle row* tomograms are midline sagittal sections through each skull. The *bottom row* tomograms are transverse sections through the skulls, enlarged $\times$2 relative to the other reconstructions. The intercommunicating middle ear cavities are in each case *shaded in red*. Note that, in *Amblysomus*, there is a greater degree of trabeculation within the middle ear cavities, including within the intercommunicating region. In *Chrysochloris*, the intercommunication between right and left ears takes the form of an open channel between the cochleae; this species has hypertrophied mallei. The *scale bar* represents 10 mm for the ventral reconstructions and sagittal tomograms, but 5 mm for the transverse tomograms

**Fig. 5:**
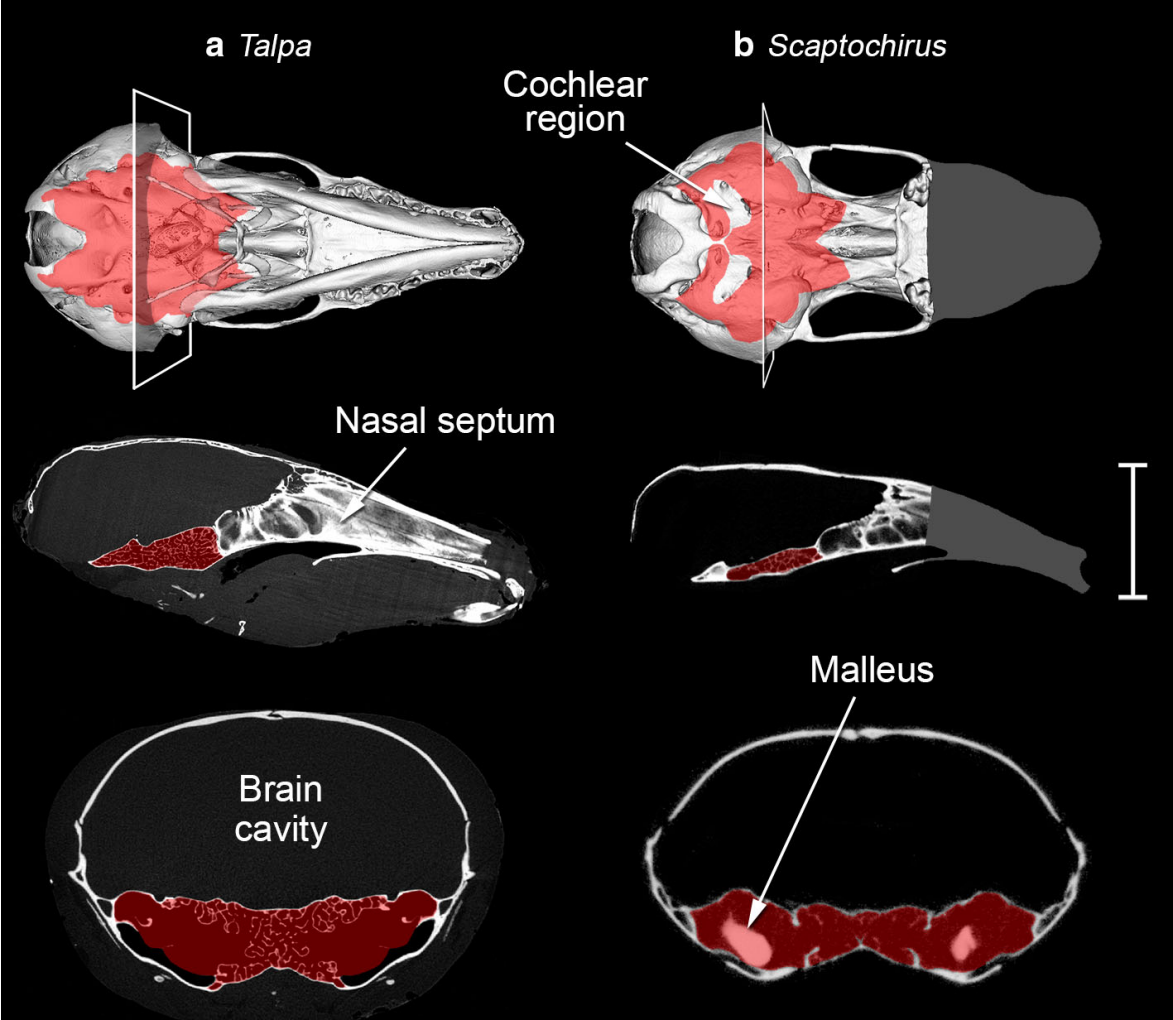
Micro-CT reconstructions of the skulls of two talpid moles, **a** *Talpa europaea*, on the *left*, and **b** *Scaptochirus moschatus*, on the *right*. The *Talpa* specimen was a skinned but otherwise undamaged head: the hyoid apparatus is intact. The *Scaptochirus* specimen was a prepared skull without the mandible. This scan did not include the snout region, the approximate extent of which is indicated by *grey shading*. The *upper row* shows Microview reconstructions of the skulls in ventral view: the planes of the transverse sections shown below are indicated. The *middle row* tomograms are midline sagittal sections through each skull. The *bottom row* tomograms are transverse sections through the skulls, enlarged $\times$2 relative to the other reconstructions. The intercommunicating middle ear cavities are in each case *shaded in red*. Both moles have trabeculated middle ear cavities; *Scaptochirus* has hypertrophied mallei. The *scale bar* represents 10 mm for the ventral reconstructions and sagittal tomograms, but 5 mm for the transverse tomograms

Basicranial bones may be pneumatised by extensions of the middle ear cavities, to the point where a complete air connection exists between right and left middle ears (Fig. 1e). So far as is known, this has occurred in only two families of living mammals, the golden moles (order Afrosoricida, family Chrysochloridae: Fig. 4) and the talpid moles (order Soricomorpha, family Talpidae: Fig. 5). These families are only very distantly related, and they evolved both their subterranean habits and interaural connections independently.

The simplest approach used by the author to determine the existence of a connection between the left and right middle ear cavities of these animals has been to add droplets of water into one cavity, whereby the extent of penetration of the liquid through the basicranium can be directly observed through the thin, translucent bone. Pressing inwards on one tympanic membrane also results in the contralateral one visibly bulging outwards (Aitkin et al. 1982; pers. obs.). The morphology is best investigated through the use of serial histological sections and computed tomography, and it is described in more detail in the next section.

## Internally coupled ears in talpid and golden moles

### Middle ear cavity intercommunication in golden moles

The first mention of middle ear intercommunication in golden moles was made by Hyrtl (1845) in his description of *Chrysochloris asiatica*. Hyrtl was convinced that the middle ears of this animal communicate by means of a sinus within the sphenoid. Forster Cooper (1928) found the same sinus in a museum specimen of “*C. tenuis*”, now regarded as the same species, but he thought that in life it was probably filled with blood vessels. Later studies confirmed the presence of an open connection in *C. asiatica* (Simonetta 1956, 1957; von Mayer et al. 1995). Middle ear intercommunication has now been found in nearly all golden mole species investigated (Table 1), the single exception being *Chrysospalax villosus* (Forster Cooper 1928; Mason 2003, 2004, 2007; von Mayer et al. 1995). Although the material available for examination has been limited, a very narrow connection does appear to be present in the only other *Chrysospalax* species, *C. trevelyani* (Mason 2007).

The nature of the middle ear intercommunication differs between species. In *Amblysomus hottentotus*, several basicranial bones are fused, pneumatised by extensions of the tympanic cavity and filled with fine, bony trabeculae, giving this region a spongy appearance. The diameter of the air channels between the trabeculae averages around 0.3 mm, but the region immediately behind the tympanic membrane remains largely free of trabeculation. Communication between the right and left middle ear cavities occurs within the trabeculated, pneumatised basisphenoid (Fig. 4a). *Neamblysomus* species have a very similar morphology (Mason 2003). In contrast, intercommunication between the two ears in *Chrysochloris asiatica* takes the form of a narrower, open channel, free of trabeculae, which extends within the basisphenoid below and between the cochleae (Fig. 4b). This channel is irregularly shaped, but of the order of 1.5 mm in diameter and 2.5 mm in length. A similar channel connecting left and right middle ears is found in *Eremitalpa* (Mason 2003; von Mayer et al. 1995).

**Table 1:** Golden and talpid mole species in which left and right middle ear cavities intercommunicate by means of air connections extending through the bones of the skull, together with the earliest known supporting references. Many species remain to be examined

Golden mole	First reference	Talpid mole	First reference
*Chrysochloris asiatica*	Hyrtl (1845)	*Talpa caeca*	Simonetta (1957)
*Eremitalpa granti*	von Mayer et al. (1995)	*Talpa* sp.	Pye and Hinchcliffe (1968)
*Chlorotalpa* spp.	von Mayer et al. (1995)	*Talpa europaea*	Aitkin et al. (1982)
*Amblysomus* and *Neamblysomus* spp.	Mason (1999)	*Scapanus orarius*	Mason (2001)
*Carpitalpa arendsi*	Mason (2004)	*Scapanus townsendii*	Mason (2006)
*Calcochloris obtusirostris*	Mason (2007)	*Parascaptor leucura*	Mason (2006)
*Chrysospalax trevelyani*	Mason (2007)	*Scaptochirus moschatus*	Mason (2007)
...but not in *Chrysospalax villosus* (see e.g. von Mayer et al. 1995)		...but not in others including *Scalopus*, *Condylura*, *Parascalops* and *Neurotrichus* species (see e.g. Mason 2006)

**Table 2:** Middle ear cavity volumes and midline intercommunication areas in some of the mammals examined in the present study

Species	Body mass (g)	Middle ear volume ($\hbox {mm}^{3}$)	Midline intercommunication area ($\hbox {mm}^{2}$)
*Amblysomus hottentotus*	70	91	4.9
*Chrysochloris asiatica*	50	89	2.0
*Talpa europaea*	91	207	18.7
*Scaptochirus moschatus*	80	139	7.9
*Macroscelides flavicaudatus*	34	748	-

For talpid and golden moles, which have intercommunicating middle ear cavities, the volumes are those of left and right cavities combined. For *Macroscelides*, in which the middle ears do not intercommunicate, volumes are for one cavity only, taken from Mason (2016a). The “midline intercommunication area” is the cross-sectional area of the middle ear cavity in the sagittal midline, measured from the sagittal tomograms shown in Figs. 4 and 5. Ossicular volumes have not been included in middle ear cavity volumes. However, the trabeculated bone which occupies parts of the middle ear cavities in talpid and golden moles has been included within both volumes and areas, so most of these values represent overestimates. Body masses are included for comparison: these were recorded for the *Talpa* and *Macroscelides* specimens, but are otherwise approximate species averages

Golden mole taxonomy is in a state of flux, but *Amblysomus* and *Neamblysomus* have historically been grouped within the subfamily Amblysominae, and *Chrysochloris* and *Eremitalpa* within the Chrysochlorinae (Asher et al. 2010; Bronner and Jenkins 2005). The nature of the interaural coupling in other golden mole species has not been examined in such detail, and it remains to be seen whether their morphological patterns correspond to subfamilial placement in the same way. It is possible that interaural coupling evolved only once within the common ancestors of extant golden moles, and has been secondarily lost in *Chrysospalax villosus*.

### Middle ear cavity intercommunication in talpid moles

Although certain talpid moles have long been known to have spongy basicranial bones, often compared to those of birds, communication between left and right middle ears in this group was apparently not suspected until Simonetta (1957) commented on this in relation to *Talpa caeca*. Later authors noted the same in an unspecified *Talpa *species (Pye 1972; Pye and Hinchcliffe 1968, 1976), and then in *Talpa europaea* (Aitkin et al. 1982).

In *Talpa europaea* (Fig. 5a), the basisphenoid, basioccipital and ventral alisphenoid bones are fused, pneumatised, and appear spongy and trabeculated. Petrosal and ectotympanic bones also contribute to the walls of the middle ear, but do not appear to be trabeculated. The intercommunication of the right and left middle ear cavities across the midline occurs in what are probably basisphenoid and basioccipital components, by means of numerous, narrow air channels, each averaging around 0.2 mm diameter. There is more spongy bone in this central region than there is in *Amblysomus*, and the area of intercommunication in the midline is four times greater (Table 2). The available scans of *Scaptochirus moschatus* (Fig. 5b) were of poorer resolution: an interaural connection appears to exist within the trabeculated basisphenoid but the basioccipital is not pneumatised. Caudal to the cochlea, an extension of the middle ear cavity approaches its contralateral counterpart but does not meet it. This inflated region, which is not trabeculated, appears to be contained within the petrosal bone.

Among the talpid species now known to have an interaural connection (Table 1), the genera *Talpa*, *Scaptochirus* and *Parascaptor* are all grouped in the Talpini (He et al. 2014; Sánchez-Villagra et al. 2006). It is presently unknown whether the other moles in this tribe have interconnected middle ear cavities. Moles in the North American genus *Scapanus*, placed within the Scalopini (Sánchez-Villagra et al. 2006), also have communicating middle ear cavities. Among the other scalopines, the trabeculated middle ear cavities of *Scalopus* approach each other very closely in the midline but appear to remain separated by a thin septum (Mason 2006 and see Fig. 22 in Henson 1974).

Within the Talpidae, it seems most likely that internally coupled ears evolved once within the ancestors of the Talpini, and separately within the Scalopini. Interaural connections are not found in talpids which spend more time on the surface and are hence less strictly subterranean (Mason 2006).

### Massive mallei and missing muscles

The presence of internally coupled ears in talpids and golden moles is only one of several unusual characteristics of the middle ears of these animals. Others include the markedly enlarged mallei of some genera, believed to be associated with hearing by bone conduction, and the reduction or loss of the tensor tympani muscle [see Mason (2013) for a recent review]. Given the distribution of these characteristics among species, it seems most likely that interaural communication and the loss of the tensor muscle preceded the evolution of ossicular hypertrophy, in both talpid and golden moles, but it should be noted that the ossicles are not hypertrophied in all species with an interaural connection. Whether internally coupled ears and tensor loss are a prerequisite for ossicular hypertrophy, or whether these features evolved independently, remains unclear.

### Hearing in talpid and golden moles

No behavioural audiograms have been published for talpid moles, and only limited information regarding their hearing capabilities exists. Kriszat (1940) trained a captive *Talpa europaea* to respond to the pure tones of a flute between 0.25 and 3.5 kHz, but he did not test frequencies outside of that range. Aitkin et al. (1982) found that behavioural responses could be elicited at frequencies from 0.2 to 15 kHz in the same species, but low speaker output may have limited responses at higher frequencies. The best sensitivity was between 5 and 8 kHz but sound pressures of 80 dB SPL were required, suggesting that the hearing is relatively insensitive. Sound-evoked responses were recorded from the inferior colliculus over a similar range. Aitkin et al. noted that the moles sometimes oriented their heads towards the speakers, which implies that they could localise sound. The most thorough assessment of the hearing range of talpids was by Konstantinov et al. (1987), who recorded evoked potentials from the inferior colliculi in both *Mogera robusta* and *Talpa europaea*. Although they found responses to sound extending into the high ultrasonic range in shrews, Konstantinov et al. were only able to record responses at frequencies from 0.1 to 22 kHz in *Mogera*, and up to 16 kHz in *Talpa*. Maximum sensitivity was at around 3 kHz. These results suggest that the hearing of these moles is restricted to relatively low frequencies, largely within the sonic range.

It might be expected that those golden mole species with hypertrophied ossicles would also have hearing limited to a low-frequency range, because of the high mass of the malleus. However, it was observed in laser vibrometric experiments that the rotatory axis of the enlarged ossicles of *Chrysochloris* changes at airborne sound frequencies above around 200 Hz, such that the ossicles start to vibrate in a mode which minimises inertia (Willi et al. 2006). Resonance was observed at the tip of the long process of the incus at frequencies from 1.3 to 2.2 kHz. Responses dropped off after that but were still observed at 10 kHz, the highest frequency tested. The audition of golden moles has not been experimentally investigated beyond this, and the upper limits to their hearing remain unknown.

### The experimental study of Coles et al. (1982)

The only published experimental investigation of acoustic coupling through the heads of moles was performed by Coles et al. (1982). They removed the tympanic membrane from one ear of each of seven moles (*Talpa europaea*), and put a microphone in that position. They then played sound from a loudspeaker adjacent to the contralateral ear, and recorded the sound intensity. They found good transmission (under 6 dB attenuation) at frequencies from 0.5 to 6 kHz, but increasing attenuation after that. Blocking the external meatus nearer to the speaker resulted in severe attenuation of 16-40 dB for most frequencies tested. Sound transmission was in this case comparable to that recorded in a rat, which lacks internally coupled ears. They also found evidence of phase differences between the sound pressures on either side of the moles’ eardrums, which were dependent on speaker position.

The animals examined by Coles et al. (1982) must presumably have been dead, so post-mortem effects such as drying of tympanic membranes, as well as the invasive surgery required to get the microphone into the position of the eardrum, would be expected to have affected the frequency responses recorded. Bearing in mind these limitations, the Coles study remains the only published report to demonstrate directly that *Talpa* can, potentially, use its tympanic membranes as pressure-difference receivers, which might allow it to localise low-frequency sound sources.

## Discussion

Although there are several anatomical routes by which sound from one ear could in principle reach the contralateral ear, the strength of acoustic coupling in mammals is in most cases unlikely to be enough to give the tympanic membrane significant directionality. For example, chinchillas have hypertrophied bullae which approach each other closely in the midline and also semi-patulous Eustachian tubes, but there is no evidence that their middle ears are acoustically coupled to any significant extent. Experimental data are lacking in the cases of the sengi *Macroscelides* and the talpid mole *Scalopus*, which have cavities separated by a thin septum only, and in shrews, which possess soft tissue pathways between their middle ears. Shrews are known to have good high-frequency hearing, so from this point of view they would appear not to require pressure-difference sound localisation.

Internal acoustic coupling is more likely to be significant in those species with a complete air passage between right and left ears, because this is likely to conduct sound with less attenuation. An air connection may be achieved via open communication between middle ear and pharynx, reportedly the case in the platypus (Figs. 1d, 3), or via pneumatisation of the basicranial bones, as in certain talpid and golden moles (Figs. 1e, 4, 5). Coles et al. (1982) have shown that good acoustic coupling does indeed occur in the mole *Talpa*, an animal which appears to have hearing limited to sonic frequencies. The hearing abilities of golden moles and the platypus have not been well studied, but the indications are that their hearing may also be restricted to relatively low frequencies. These animals, which all lack pinnae, might well benefit from the sound localisation possibilities afforded by internally coupled ears.

The wide communication between middle ear and nasopharynx in *Ornithorhynchus* is considered by Zeller (1993) to be a derived condition. However, if the monotreme middle ear apparatus evolved separately to that of therians (Rich et al. 2005), it is possible that the platypus actually retains a primitive (pre-mammalian) pharyngo-tympanic connection, and that echidnas, which have narrow Eustachian tubes, are the derived monotremes in this respect. This deserves further investigation, based on more detailed studies of the pharyngeal region in these animals.

### Evolution of interaural coupling via the basicranium

Expansion of middle ear cavities is expected to improve low-frequency hearing by reducing acoustic stiffness (Ravicz and Rosowski 1997). This could well benefit a subterranean mammal, given that airborne sound of a few hundred Hertz propagates better than higher-frequency sound in tunnels (Heth et al. 1986; Lange et al. 2007). Expansion of ear cavities towards the midline might reflect spatial constraints on how prominent the bullae can be ventrally (Fleischer 1978). The pneumatisation of the basicranium in moles and golden moles may therefore have been driven by the resulting improvement in low-frequency audition. However, the basicranium is quite flattened in moles and, despite its pneumatisation, the overall middle ear cavity volume is not exceptionally large (Mason 2001; Table 2). It is therefore possible that the pneumatisation evolved, at least to begin with, not for hearing at all but to improve the mechanical properties of the basicranium: a hollow structure should maintain high strength with minimal investment of bony material (Kolmer 1913).

**Fig. 6:**
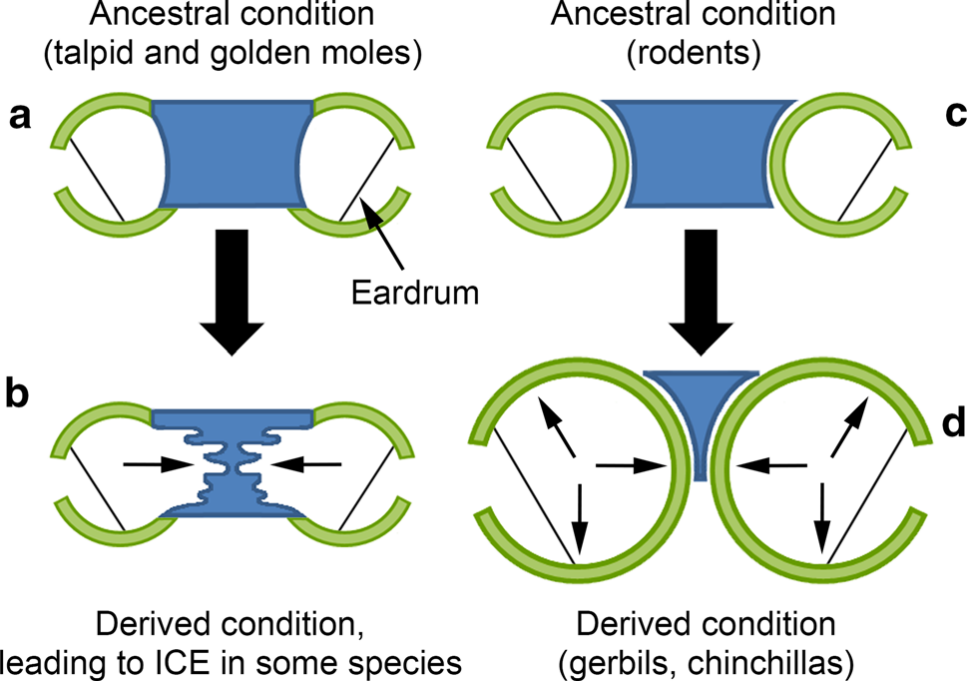
Proposed evolutionary pathways of middle ear cavity expansion in mammals. *Small arrows* indicate the directions of expansion. **a** Schematic transverse section through the basicranial bones of ancestral talpid and golden moles. Middle ear cavity walls include a midline basisphenoid element, in *blue*, and other bony elements, in *green* (bullae are actually incomplete in the most primitive talpid moles). **b** The derived condition in talpid and golden moles is that the basisphenoid has become pneumatised. This excavation has proceeded in some groups to the point that the two middle ear cavities join together in the midline, leading to internally coupled ears (ICE). **c** Schematic transverse section through the basicranial bones of an ancestral rodent. The basisphenoid does not contribute to the bullar walls. **d** In many rodents which inhabit arid environments, such as gerbils and chinchillas, the auditory bullae have become enlarged so as to improve low-frequency audition. As part of this enlargement, the bullae approach each other at the midline, but they remain separated

The walls of the middle ear cavity are composed of different bony elements in different mammalian groups. One potential contributor is the basisphenoid, which is an unpaired, midline bone: talpid moles, golden moles and sengis are among the few mammalian groups to have a basisphenoid component to their middle ear cavity walls (Mason 2016a; Novacek 1977; Van der Klaauw 1931). In *Talpa*, pneumatisation seems to have spread from the basisphenoid into the basioccipital, another midline element. If the middle ear cavity has a midline component to its medial wall, it has the potential to approach its contralateral counterpart very closely, to the point where the two cavities are separated by a thin septum (*Scalopus*, *Macroscelides*), or not separated at all, as in certain talpid and golden moles (Fig. 6a, b).

Like most other mammals, rodents lack a basisphenoid contribution to their middle ear cavity walls. In those with very expanded bullae, such as gerbils, the ectotympanics closely approach each other at the midline but remain separated from their contralateral counterparts (Mason 2016a; Fig. 6c, d). No rodent species is known to have intercommunicating cavities, so it is no surprise that subterranean rodents, which have very restricted high-frequency hearing, have poor sound-localising abilities (Heffner and Heffner 1990, 1992b, 1993). Being insectivorous, talpid and golden moles may have a greater need for accurate sound localisation.

The nature of the cavity intercommunication varies among talpid and golden moles from a simple channel to multiple, maze-like pathways through trabeculated bone, and the extent of the intercommunication at the midline of the skull varies (Table 2). The functional differences between these morphologies, if any, remain unknown.

## Conclusion

The ears of most mammals are believed to be acoustically independent. However, the right and left middle ear cavities of the platypus and those of certain talpid and golden moles are linked by patent air pathways, leading to the prediction that their tympanic membranes should act as pressure-difference sound receivers over a certain frequency range. Whether these animals actually make use of this in pressure-difference sound localisation remains to be confirmed. There are at present more questions than answers, but the identification of internally coupled ears in this small collection of unusual mammals offers exciting possibilities for future research.
